# Multi-Sensor Fusion Approach for Improving Map-Based Indoor Pedestrian Localization

**DOI:** 10.3390/s19173786

**Published:** 2019-08-31

**Authors:** Hsiang-Yun Huang, Chia-Yeh Hsieh, Kai-Chun Liu, Hui-Chun Cheng, Steen J. Hsu, Chia-Tai Chan

**Affiliations:** 1 Department of Biomedical Engineering, National Yang-Ming University, Taipei 112, Taiwan; 2 Department of Information Management, Minghsin University of Science and Technology, Hsinchu 304, Taiwan

**Keywords:** indoor pedestrian localization, multi-sensor fusion, inertial sensor, light sensor

## Abstract

The interior space of large-scale buildings, such as hospitals, with a variety of departments, is so complicated that people may easily lose their way while visiting. Difficulties in wayfinding can cause stress, anxiety, frustration and safety issues to patients and families. An indoor navigation system including route planning and localization is utilized to guide people from one place to another. The localization of moving subjects is a critical-function component in an indoor navigation system. Pedestrian dead reckoning (PDR) is a technology that is widely employed for localization due to the advantage of being independent of infrastructure. To improve the accuracy of the localization system, combining different technologies is one of the solutions. In this study, a multi-sensor fusion approach is proposed to improve the accuracy of the PDR system by utilizing a light sensor, Bluetooth and map information. These simple mechanisms are applied to deal with the issue of accumulative error by identifying edge and sub-edge information from both Bluetooth and the light sensor. Overall, the accumulative error of the proposed multi-sensor fusion approach is below 65 cm in different cases of light arrangement. Compared to inertial sensor-based PDR system, the proposed multi-sensor fusion approach can improve 90% of the localization accuracy in an environment with an appropriate density of ceiling-mounted lamps. The results demonstrate that the proposed approach can improve the localization accuracy by utilizing multi-sensor data and fulfill the feasibility requirements of localization in an indoor navigation system.

## 1. Introduction

The interior space of large-scale buildings, such as hospitals, with a variety of departments, is so complicated that people may easily lose their way while visiting. Wayfinding is an important issue in a huge hospital, especially for patients who have multiple destinations in the course of a single visit [1,2]. Poor wayfinding may lead to several problems which often results in bad experiences and consequences, to not only patients but also employees [1,3]. Spending a lot of time wayfinding and arriving late for appointments can make patients feel frustrated and stressed. Unfamiliarity with the environment brings about the risk of entering restricted areas and can be hazardous to visitors. Difficulties in wayfinding cause stress, anxiety, unsafety and frustration to patients and families. Moreover, there are additional costs for hospitals as a result of poor wayfinding, as staff may be interrupted from their work in order to help people find their way.

A common strategy for solving the poor wayfinding problem is setting up instruction signs. People who visit an unfamiliar place can find a direction to their destination by reading the information on the instruction signs. However, the names of units can be similar and can, therefore, create confusion for people trying to navigate to their intended location. [4,5,6]. Therefore, guiding people to the destination is an important issue of clinical practice, since instruction signs can be unreliable. An indoor navigation system can be utilized to guide people from one place to another. An indoor navigation system includes route planning and the localization of moving subjects within interior building [7]. The localization of moving subjects is a critical-function component in indoor navigation. If the given position is inaccurate, the suggested direction and path in the route planning may be confusing for people looking to find their destination.

Since wayfinding becomes a common issue, several localization systems have been proposed in recent years, including infrared (IR), ultrasound, radio frequency identification (RFID), Bluetooth, Wi-Fi and pedestrian dead reckoning (PDR) [8,9,10,11,12,13]. IR-based localization systems calculate position based on time of arrival (TOA), e.g., the active badge system. An active badge worn on a subject transmits an infrared signal to receivers to provide information for localization [14,15]. The infrared radiation can be confined to the inside of the room because the radiation does not penetrate through walls. However, the accuracy is affected by the multipath errors and costs of the system hardware are expensive [12]. Ultrasound systems utilize time of arrival (TOA) and time difference of arrival (TDOA) to estimate position by measuring the distance between the station and mobile devices [16,17,18]. Although ultrasound-based localization systems can achieve a better accuracy compared to IR-based localization system, the ultrasound wave can be interfered with by the reflection of ultrasound signal when it collides with metals [19]. The radio frequency-based localization system such as RFID, Bluetooth and Wi-Fi can estimate position by measuring distance between transmitters and receivers based on triangulation, proximity and fingerprinting methods [20,21,22,23,24,25,26,27,28]. However, signal propagation in an indoor environment may be interfered with by various obstacles and electronic devices [12]. PDR systems utilize inertial sensors such as accelerometers and gyroscopes to calculate the position based on a previously determined position, estimated step information and heading direction [29,30,31]. The main advantages of PDR systems are that there is no additional infrastructure required and avoiding radio frequency interference [32]. Considering the indoor environment of hospitals that may have many electronic devices, PDR systems are more suitable than other systems for developing indoor localization. Nevertheless, there are two issues in PDR systems. Firstly, the determination of the initial position is important for PDR systems [9]. The estimated position cannot reach high accuracy with an improper initial position. To overcome the limitation, the literature focuses on providing the initial position for the PDR system using near field communication (NFC) [33] and augmented reality [34,35,36]. Secondly, the localization error may accumulate within the walking path [29]. The PDR systems can provide high localization accuracy in a short-range. However, drift error and incorrect estimation of step length can lead to an accumulative of errors, since the current position is estimated based on previous position. Jo Agila et al. [37] proposed an indoor navigation system called Footpath to deal with drift error of heading estimation. By matching the heading direction with the orientation on the known route, Footpath can reduce inaccuracy at corners. Although the drift error of heading estimation is solved, the incorrect step length may cause an accumulative error on a straight path.

To improve the accuracy of indoor localization, combining multi-sensor data is one of the solutions. Pedestrian dead reckoning is a technology that is widely employed for multi-sensor fusion, due to the advantage of being infrastructure-free. However, the accuracy of the PDR system is influenced by accumulative error. To overcome the accumulative error of the PDR system, several researchers combine different technologies to compensate for the disadvantage of each technology. There are three main localization systems commonly combined with the PDR system, including Wi-Fi, Bluetooth and light sensors. Since Wi-Fi routers are widely deployed in public environments, Wi-Fi is one of the preferred technologies for combined systems. Zhenghua Chen et al. [38] utilized a Kalman filter and landmarks to estimate the position by using the Wi-Fi fingerprinting approach and the PDR system. Frederic Evennou et al. [39] combined information from inertial sensors and Wi-Fi by using a particle filter to improve the positioning accuracy. Huaiyu Li et al. [40] utilized a trusted point determination algorithm to integrate the position from the inertial sensors and the Wi-Fi fingerprinting approach. Lyu-Han Chen et al. [41] employed a maximum likelihood-based fusion algorithm to fuse a PDR system with a fingerprint scheme using Wi-Fi. Taking into account the power consumption, several researchers utilize the Bluetooth capabilities of modern smartphones to develop the fusion localization. Xin Li et al. [42] proposed a fusion positioning approach based on an extended Kalman filter. The extended Kalman filter estimated the position by integrating information from the PDR system and the fitting curve of Bluetooth. Jinglong Li et al. [43] developed a support vector machine (SVM) classification algorithm based crowdsourcing method to generate Bluetooth landmarks and applied a particle filter to combine the position from the landmarks detection and the PDR system. Zhenghua Chen et al. [44] utilized a weighted pass loss model to determine the initial position from Bluetooth and Wi-Fi. Then, an extended Kalman filter was applied to estimate the position from the information of inertial sensors and Bluetooth attached on the walls. To reduce the cost of infrastructure, the light sensor is a promising technology for localization in recent years [45]. The light sensor embedded into a smartphone can detect received light intensity while pedestrians pass through a luminary in the indoor environment. With the information of received light intensity, many algorithms can be utilized for localization, including triangulation and fingerprinting techniques. Owing to characteristics of low cost and a long lifetime, some literature focuses on developing light sensor-based fusion approaches. Qiang Xu et al. [46] proposed an indoor localization system which utilized a particle filter to combine information from inertial sensors and a light sensor on a smartphone. A luminary-assisted stride length estimation method was developed to obtain the instantaneous stride length. Antonio Jimenez et al. [47] improved pedestrian dead reckoning systems with the information provided by light matching. The light matching approach estimated the localization and heading direction by a particle filter. While pedestrians passed below the luminaries, the number of detected lights and the walking distance of pedestrians were utilized to update the particles. Muhammad Yasir et al. [48] developed an indoor positioning system using a light sensor and accelerometer. The received light intensity from the light sensor and orientation angles from the accelerometer were applied to estimate the distance between the light sensor and the light-emitting diode (LED) transmitter, and thus calculate the localization of pedestrians.

This study aims to improve the performance of the indoor pedestrian localization system using combined information from inertial sensors, a light sensor and the Bluetooth embedded in a smartphone. A multi-sensor fusion approach is proposed to deal with the issue of accumulative error and provide accurate position information. Firstly, the information from inertial sensors can estimate the position by using step and heading information. Secondly, the light sensor signal combines with step detection to detect the occurrence of light events, which can be detected as the pedestrian passes under a ceiling-mounted lamp. Finally, to tackle the accumulative error, the pedestrian position, step length and heading direction are modified by the location information of the corresponding ceiling-mounted lamp light information, obtained from a pre-surveyed lamp map. This proposed multi-sensor fusion approach uses the map information to alleviate the issues of conflicting evidence [49,50] or divergence measure [51,52] in multi-sensor fusion. The localization is calculated by PDR or FirstFit approach if there is no light event detected. When a light event is detected, the localization is modified to the absolute position derived by the lamp position. Therefore, the localization is estimated by either PDR/FirstFit or lamp position. The multi-sensor fusion utilizes absolute localization information from light rather than a weighted combination to estimate localization. The contributions of this paper are as follows:

- The multi-sensor fusion approach, utilizing inertial sensors, a light sensor and Bluetooth embedded in a smartphone, requires less infrastructure in the environment and prevents the interference of radio signals involved in the indoor environment.
- The multi-sensor fusion approach can improve the accuracy of indoor pedestrian localization. The issue of the accumulative error can be overcome by the information from the light sensor and Bluetooth. Since pedestrians may follow the planning route of the navigation system to the destination, the walking route is restricted to a known planning route. The actual position and orientation of pedestrians can be obtained from examining the ceiling-mounted lamps passed by pedestrians.
- The actual position information from the light sensor and Bluetooth can provide individual information for modifying the step length and heading information. The individual step length can be modified by the distance between two ceiling-mounted lamps and the heading direction can be reset by the known planning route from the map.

The rest of this work is organized as follows: In Section 2, we introduce the proposed multi-sensor fusion approach, including data acquisition, the motion model, the light model and the decision making. The experiment protocols are described in Section 3. In Section 4, the experimental results are presented and the performance of the proposed multi-sensor fusion approach is demonstrated. The effect and potentiality of the proposed approach are discussed in Section 5. Finally, the conclusion of the proposed multi-sensor approach is presented in Section 6.

## 2. Multi-Sensor Fusion Approach

**Figure 1:**
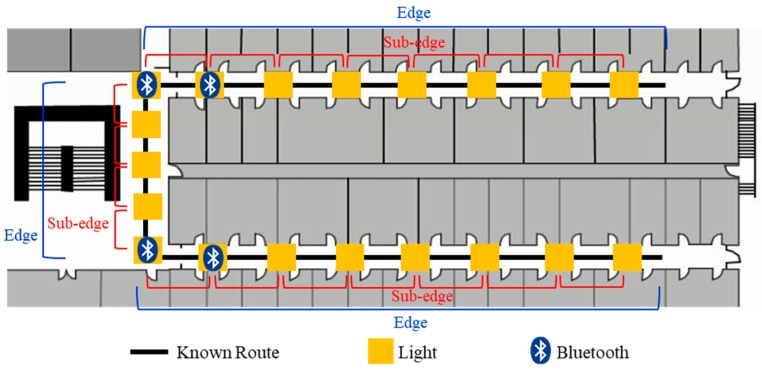
An example of Bluetooth and lamps deployment in a known route. An edge indicates the straight path of the route and a sub-edge represents the segment between two lamps.

Developing an accurate and robust indoor localization system is still a challenging problem. To improve the accuracy of the localization system, combining different technologies is one of the solutions. Therefore, we propose a multi-sensor fusion approach to improve the accuracy of the PDR system by utilizing a light sensor, Bluetooth and map information. Since the walking route of the pedestrian can be known by the planning route from the navigation system, the localization can be inferred by detecting lamps and pre-installed Bluetooth beacons that the pedestrian passes and comparing the detected information to pre-surveyed map information which includes lamps and beacons localization in the environment. As an example shown in Figure 1, the known walking route can be divided into three parts by corners called edges. Then, each edge can be segmented into several sub-edges by lamps. In the fusion approach, we can modify the localization of PDR system by edge information from Bluetooth and sub-edge information from the light sensor.

**Figure 2:**
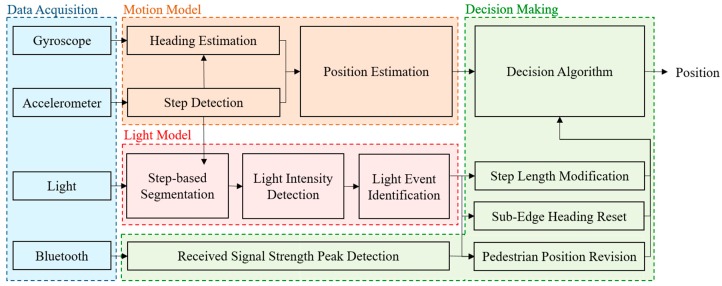
The system architecture of the proposed multi-sensor fusion approach.

The system architecture of the proposed multi-sensor fusion approach is shown in Figure 2. The approach is divided into four functional components, including data acquisition, motion model, light model and decision making. In the data acquisition, an accelerometer, a gyroscope, a light sensor and Bluetooth embedded in a smartphone are employed to develop the proposed multi-sensor fusion approach. The motion model utilizes the accelerometer and gyroscope to obtain movement information, such as step and orientation. The light model detects the occurrence of light events by the light sensor and step information. In the decision making, the information from the motion model, light model and Bluetooth are combined to compensate for the accumulative error and improve the accuracy of the PDR system.

### 2.1. Data Acquisition and Preprocessing

There are four sensors utilized in this approach, including the accelerometer, gyroscope, Bluetooth and light sensor. All of these sensors are embedded in a hand-held smartphone which is facing up during walking to collect the pedestrian’s movement information. The accelerometer and gyroscope are utilized to estimate the pedestrian position by a traditional PDR approach. Bluetooth and the light sensor recognize the light event while pedestrians pass under ceiling-mounted lamps. In data preprocessing, the collected data of these sensors is resampled with 30.3 Hz at first. Then, we exploit a low-pass filter with a cutoff frequency of 8.95 Hz to the resampled data for noise reduction. The filtered data is transferred to the motion model and the light model for further position estimation.

### 2.2. Motion Model

The filtered data of the accelerometer and gyroscope are utilized to track pedestrian movement. There are three function components in the motion model: step detection, heading estimation and position estimation.

**Figure 3:**
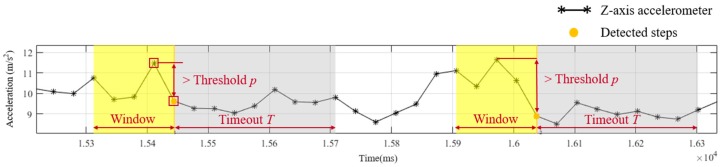
The illustration of step detection by threshold *p* and timeout *T* with a window size of five sampling points.

Firstly, step information is identified by step detection using the tri-axial accelerometer. Jiggling of a smartphone caused by body fluctuation during walking influences the z-axis acceleration. The jiggling pattern of z-axis acceleration can be utilized to detect step information. A sliding window technique and threshold-based identification are applied to obtain the number of walking steps [37]. The sliding window technique with a window size of five sampling points (165 ms) and 80% overlapping is utilized to segment the filtered data from data acquisition process, as Figure 3. The segmented data is identified by threshold-based identification to detect steps. In the threshold-based identification, a step event is detected if a range from the minimum to maximum of z-axis acceleration exceeds a certain threshold *p*. When a step is detected, a timeout *T* is set in the identification to prevent false step detection within a short time after the detected step. Because this study focuses on the influence of the multi-sensor fusion approach, the threshold and timeout of individual subjects for step detection are adjusted to ensure no false step detection.

Secondly, heading estimation obtains the heading orientation of each detected step by a single tri-axial gyroscope. With the z-axis angular velocity, the heading orientation can be calculated by cumulating the rotation angles from the measured angular velocities. Therefore, the heading direction $\theta_{n}$ at *n*th step is computed based on integration of z-axis angular velocities $\omega_{z,k}$ at sampling time *k* over time interval of walking steps [$t_{n-1},\;t_{n}]$ and the previous heading direction $\theta_{n-1}$ at (*n*-1)th step, which is estimated in the same manner from the heading direction $\theta_{n-2}$. Since the collected data is resampled to 30.3 Hz, the duration of sampling time *k* is 33 ms. The formula of heading estimation is shown in Equation (1).

(1) $$\theta_{n}=\theta_{n-1}+\int_{t_{n-1}}^{t_{n}}\omega_{z,k}dk.$$

Finally, position estimation integrates the information from step detection and heading estimation to calculate the pedestrian’s localization. There are two approaches applied in the position determination, including the traditional PDR and Footpath approaches. In the traditional PDR system, current position $\left(x_{n},\;y_{n}\right)$ at *n*th step can be calculated by previous position $\left(x_{n-1},\;y_{n-1}\right)$ at (*n*-1)th step, individual step length Δl and estimated heading orientation $\theta_{n}$ at *n*th step, which is defined by Equation (2). The initial position and orientation are assumed to be known. Moreover, the step length is the average step length of individual subjects measured in the experiments.

(2) $$\left[\begin{array}{c} x_{n}\\ y_{n} \end{array}\right]=\left[\begin{array}{c} x_{n-1}+\Delta l\times cos\theta_{n}\\ y_{n-1}+\Delta l\times sin\theta_{n} \end{array}\right]$$

The Footpath approach utilizes a FirstFit algorithm to compare heading direction of each step with orientation of an expected route that pedestrians follow to reach the destinations [37]. In the FirstFit algorithm, if the difference between the estimated heading direction and expected orientation is less than a certain threshold *d*, a directly matching event is triggered and position is detected by steps along the same direction. In contrast, while the difference exceeds the threshold *d* and lasts over five consecutive steps, a lookahead matching mode is activated to find a matching direction in the next edge. With the matching algorithm, Footpath has the ability to reset the location. Both localizations obtained from the traditional PDR and Footpath approaches are further processed in the decision-making function.

### 2.3. Light Model

**Figure 4:**
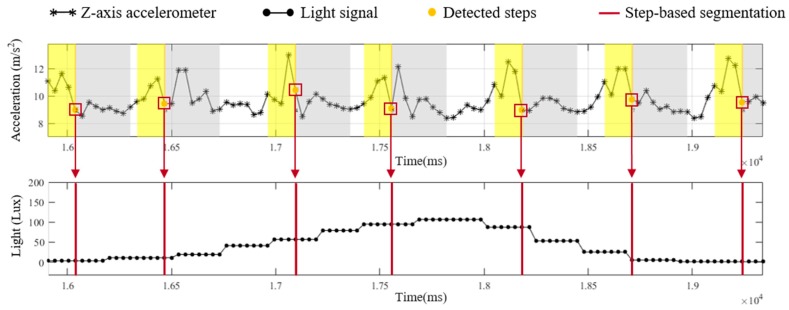
The illustration of step-based segmentation.

**Figure 5:**
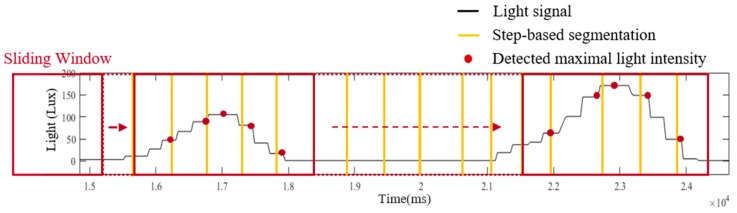
The illustration of light intensity detection with a window size of five sampling points.

In the light model, a light sensor embedded in a smartphone is exploited to provide information about the passing lamps while a pedestrian walks through a known route. The information from the light model can be utilized to modify the localization of the motion model. There are three functions implemented in the light model, including step-based segmentation, light intensity detection and light event identification. In step-based segmentation, the light signal is segmented into fragments based on the step information from motion model, as shown in Figure 4. To detect the maximal received light intensity of each step, a sliding window technique with window size of five sampling points (165 ms) and 80% overlapping is applied in the step-based light segments, as illustration of Figure 5.

**Figure 6:**
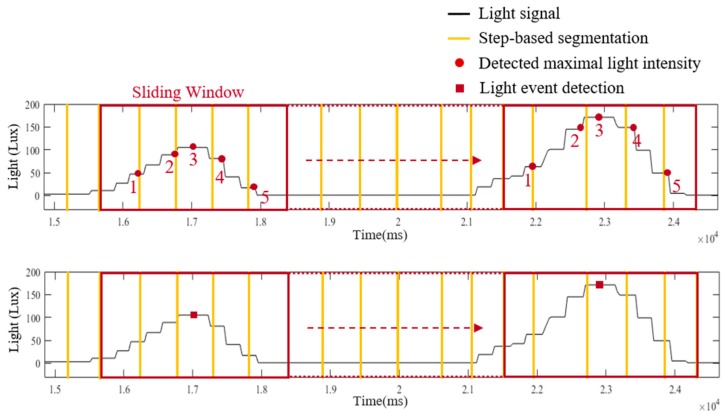
The illustration of light event identification with a window size of five steps.

With the maximal light intensity of each step from step-based segmentation and light intensity detection, the occurrence of the light event represents the pedestrian walking under a ceiling-mounted lamp and can be recognized in the light event identification. Figure 6 illustrates details about the light event identification. The detected maximal light intensity of each step is performed by a sliding window technique. For a fluorescent lamp with a length of 1 m, the range of receiving lamplight is approximately 3 m. Therefore, pedestrians need to take five steps to walk through the range of the lamplight (the average step length is 63 cm in this study). Since pedestrians need about five steps to pass through the range of the lamplight, the window size of the sliding window technique is set to five steps and the overlap is set to four steps. If the third step of a window received the maxima light intensity than other values in the window, a light event is identified and its occurrence time point is recorded. The detected light events and the occurrence time points are applied to the decision-making functional component to reduce the accumulative error caused by incorrect step length estimation and heading estimation in the PDR and Footpath approaches.

### 2.4. Decision Making

Decision making functional component fuses the information from the motion model, light model and Bluetooth to determine the final position. If there is no light event detected, the estimated position of the motion model from the PDR or FirstFit is regarded as the output position. On the contrary, while a light event is detected, the output position is modified to the position derived from the light, Bluetooth and map information.

There are two procedures for obtaining the position information. Firstly, Bluetooth is utilized to recognize the current edge information in the route. As an example shown in Figure 1, we define an edge as a straight path in a route and a sub-edge as the segment between two lamps. Bluetooth beacons are attached to the ceiling-mounted lamps close to corners for providing both the ending and beginning of the edge information in the map. The smartphone measures the received signal strength indication (RSSI) from the attached beacons. As the distance between a smartphone and a beacon decreases, the corresponding RSSI should increase. Therefore, a peak detection algorithm is utilized to detect whether a pedestrian is passing through the lamp attached with a Bluetooth beacon. If a peak is detected, the current edge information can be recognized by matching the universally unique identifier (UUID) of the Bluetooth beacon. Secondly, after verifying the current edge that the pedestrian is walking on, the identified light event while the pedestrian passing through a ceiling-mounted lamp can provide actual position information by matching the light arrangement of the edge in the map.

**Figure 7:**
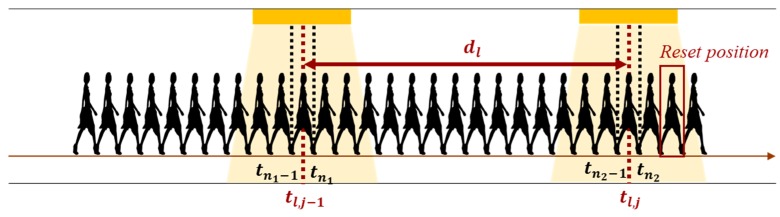
The illustration of step length modification.

After attaining the position information, the reckoning position can be revised by step length modification, sub-edge heading reset and pedestrian position revision. In the beginning, the step length is modified based on step counts and distance between two lamps [46]. Figure 7 shows the illustration of step length modification. Let $\left\{t_{l,j}|j=1,2,\ldots ,J\right\}$ denote the occurring time of light events and $\left\{t_{n}|n=1,2,\ldots ,N\right\}$ represent the detected time of step. While the pedestrian walks through the (*j*-1)th lamp within (*n*_1_-1)th to *n*_1_th steps, we can define the time of the light event at (*j*-1)th lamp as $t_{l,j-1}$ and the time of detected step as $t_{n_{1}-1}$ and $t_{n_{1}}$. After a while, the pedestrian goes through the next lamp at time $t_{l,j}$ within (*n*_2_-1)th to *n*_2_th steps, detected at time $t_{n_{2}-1}$ and $t_{n_{2}}$, respectively. The step counts between (*j*-1)th lamp and *j*th lamp can be estimated by Equation (3). The individual step length $\Delta l^{\prime }$ can be modified by step counts c and distance between two lamps $d_{l}$, as Equation (4). Next, the drift error of gyroscope can be eliminated by sub-edge heading reset. According to the identified light event in the sub-edge, the heading estimation can be reset to the orientation of the sub-edge. With the modified step length, heading direction and the actual position, the current position can be revised. Because the light event is detected when the third step of the sliding window receives the maxima light intensity, there may be two steps delayed at the light event identification. Therefore, the pedestrian position after two steps of light event $\left(x_{n_{2}+2},\;y_{n_{2}+2}\right)$ is revised by the actual position of the *j*th lamp $\left(x_{l,j},\;y_{l,j}\right)$, the modified step length Δl′ and heading direction $\theta_{l,j}$ at *j*th lamp.

(3) $$c=\frac{t_{n_{1}}-t_{l,j-1}}{t_{n_{1}}-t_{n_{1}-1}}+\left(t_{n_{2}}-t_{n_{1}}-1\right)+\frac{t_{n_{2}}-t_{l,j}}{t_{n_{2}}-t_{n_{2}-1}}$$

(4) $$\Delta l^{\prime }=\frac{d_{l}}{c}$$

## 3. Data Collection and Experiment Protocols

**Figure 8:**
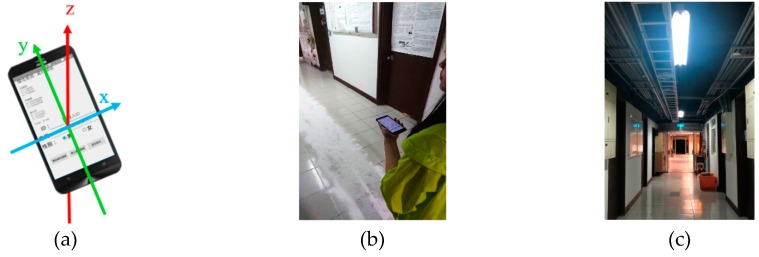
The illustration of experimental protocols. (**a**) The orientation of smartphone. (**b**) The holding manner of the smartphone. (**c**) The ceiling-mounted lamps in the experimental environment.

A smartphone ASUS Z00Ld embedded with tri-axial accelerometer, tri-axial gyroscope and light sensor is utilized to collect the motion and light information during walking. Bluetooth beacons, AprilBeacon, published by Beijing April Brother Technology Co., Ltd., are attached to the ceiling-mounted lamps to provide information for recognizing the edge of the detected light in the map. Nine subjects are recruited in this study (5 males and 4 females, 25.2 ± 1.9 years, height = 166.7 ± 6.6 cm, weight = 68.2 ± 12.0 kg). Each subject is requested to hold the smartphone in the left hand and walk on the prescribed route three times, as shown in Figure 8. Powder on the ground is applied to obtain the ground truth during walking periods.

**Figure 9:**
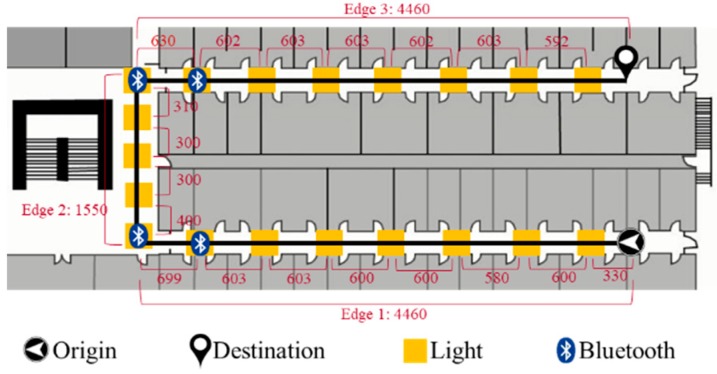
The illustration of the prescribed walking route. (Unit: cm).

**Figure 10:**
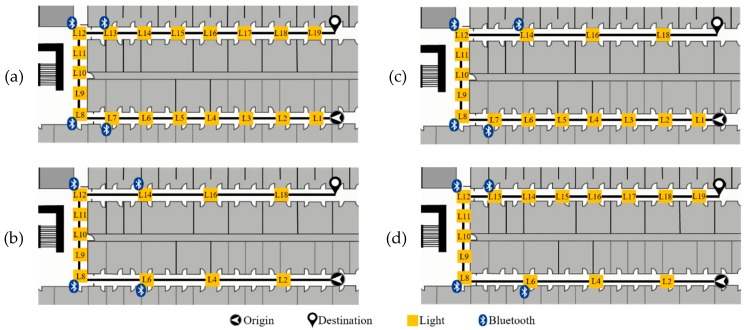
Light arrangements within the walking route. (**a**) Case 1, (**b**) Case 2, (**c**) Case 3 and (**d**) Case 4 of light arrangements are applied to evaluate the performance of multi-sensor fusion approach. (L1 to L19 represents lamp indices).

The walking route is illustrated in Figure 9. The total distance of the prescribed route is 140.7 m. The initial position and heading direction are assumed to be known. The route from origin to destination is composed of three parts, including edge 1 (origin position to the first corner), edge 2 (the first corner to the second corner) and edge 3 (the second corner to destination). In consideration of different environments, we designed four different light arrangements to evaluate the influence of different luminary density on the system performance, as demonstrated in Figure 10. Case 1 is an environment with enough light density and the sub-edge distance between ceiling-mounted lamps in case 1 is smaller than that in case 2. In addition, the luminary arrangement may not be symmetrical in the environment. Case 3 and case 4 simulate an asymmetric light arrangement of edge 1 and edge 3. To recognize the edge information, four beacons are attached on the lamps between corners. The sequence of L1 to L19 denotes the indices of passing lamps in the experiments.

## 4. Results

**Figure 11:**
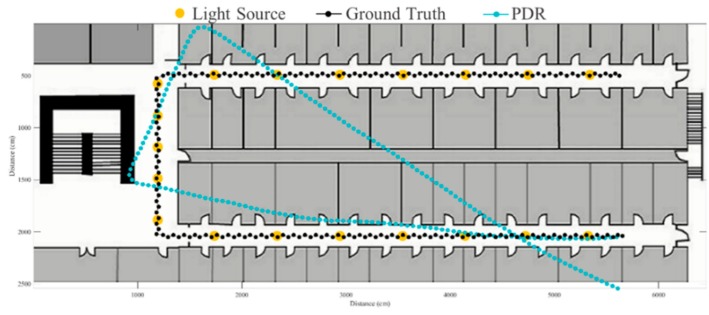
An example of localization results by the pedestrian dead reckoning (PDR) approach.

**Figure 12:**
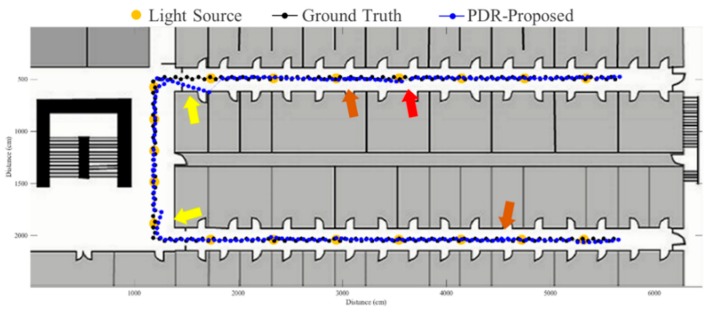
An example of localization results by proposed approach based on PDR in case 1 light arrangement. (yellow arrow: drift error; red arrow: underestimated step length; brown arrow: overestimated step length).

**Figure 13:**
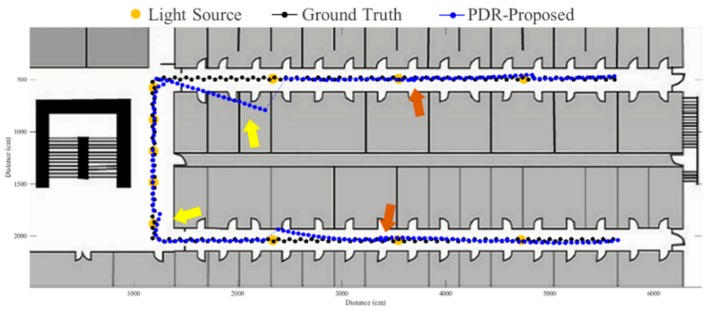
An example of localization results by proposed approach based on PDR in case 2 light arrangement. (yellow arrow: drift error; red arrow: underestimated step length; brown arrow: overestimated step length).

**Figure 14:**
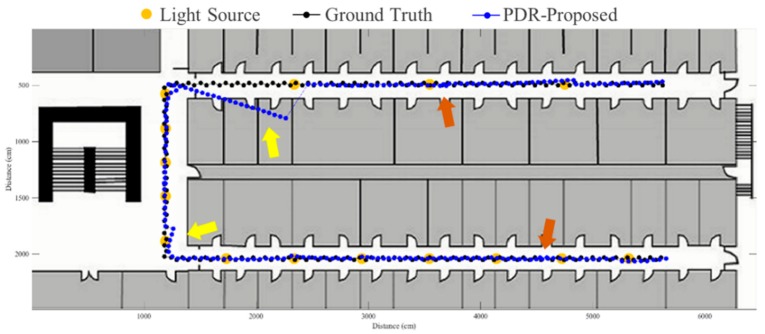
An example of localization results by proposed approach based on PDR in case 3 light arrangement. (yellow arrow: drift error; red arrow: underestimated step length; brown arrow: overestimated step length).

**Figure 15:**
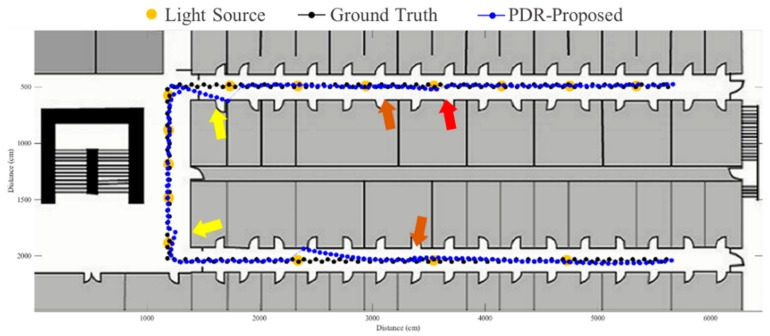
An example of localization results by proposed approach based on PDR in case 4 light arrangement. (yellow arrow: drift error; red arrow: underestimated step length; brown arrow: overestimated step length).

**Figure 16:**
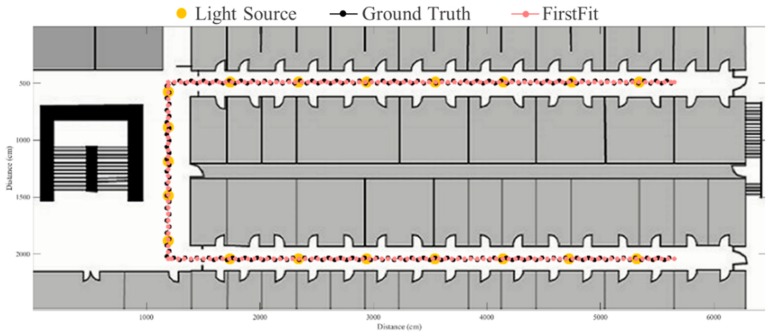
An example of localization results by FirstFit approach.

**Figure 17:**
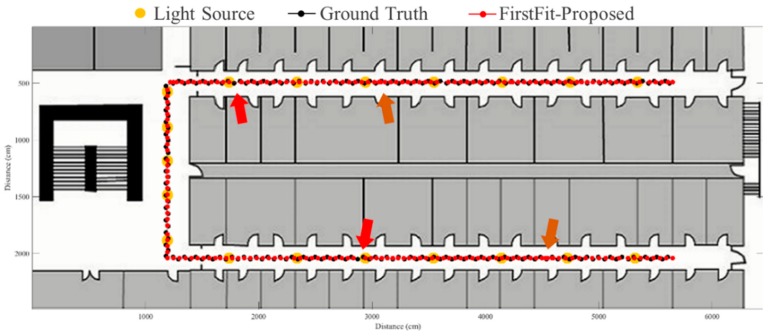
An example of localization results by proposed approach based on FirstFit in case 1 light arrangement. (red arrow: underestimated step length; brown arrow: overestimated step length).

**Figure 18:**
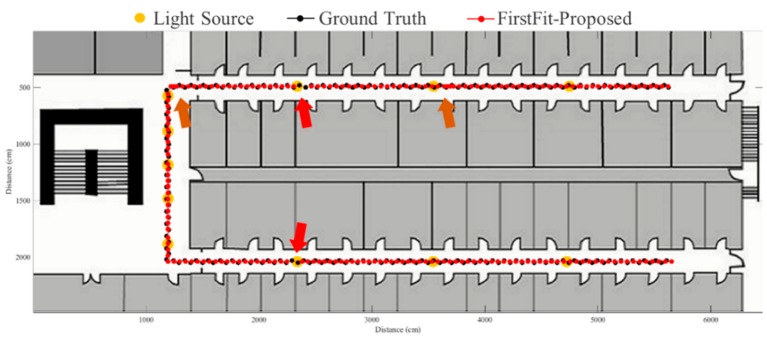
An example of localization results by proposed approach based on FirstFit in case 2 light arrangement. (red arrow: underestimated step length; brown arrow: overestimated step length).

**Figure 19:**
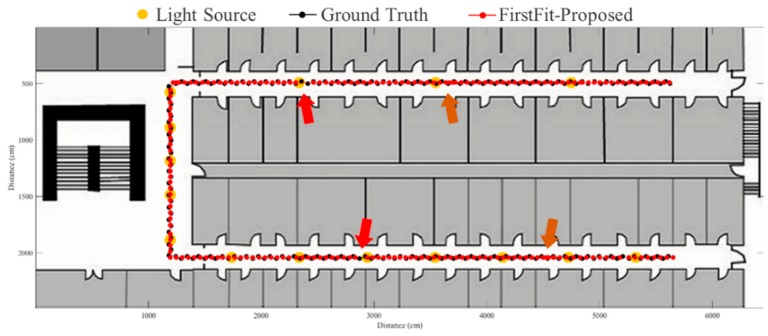
An example of localization results by proposed approach based on FirstFit in case 3 light arrangement. (red arrow: underestimated step length; brown arrow: overestimated step length).

**Figure 20:**
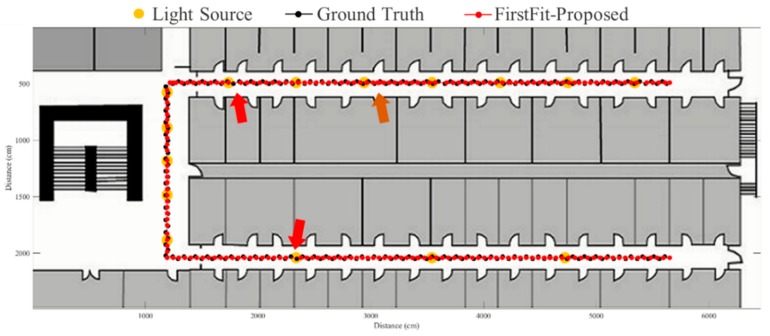
An example of localization results by proposed approach based on FirstFit in case 4 light arrangement. (red arrow: underestimated step length; brown arrow: overestimated step length).

To evaluate the proposed fusion approach, performance comparisons are made between the traditional PDR, FirstFit and proposed approaches in this study. The PDR approach utilizes the accelerometer and gyroscope of a smartphone to estimate step and heading information for localization. The FirstFit approach combines a traditional PDR with map information to reduce the drift error caused by the gyroscope. Localization results of a subject are demonstrated in Figure 11, Figure 12, Figure 13, Figure 14, Figure 15, Figure 16, Figure 17, Figure 18, Figure 19 and Figure 20. In Figure 11, the error distance of the PDR approach increases with the step number due to the drift error and incorrect heading direction. The results of the proposed fusion approach based on PDR are shown in Figure 12, Figure 13, Figure 14 and Figure 15. As with the case 1 light arrangement shown in the Figure 12, the drift error and incorrect step length can be modified by fusing multi-sensor data. Whether the step length is overestimated (brown arrow) or underestimated (red arrow), the accumulative error can be eliminated with the position revision and the individual step length modification while the subject passes through a ceiling-mounted lamp. Moreover, the drift error (yellow arrow) can be corrected by the sub-edge heading reset. While the subject turns at the corner, the drift error may affect estimation of heading direction and lead to false position estimation. With Bluetooth beacons attached on the corner, the proposed approach can update the edge information while the subject walks to the next edge. By the light information among the edge, the localization can be revised and the heading orientation can be reset to the direction of the edge. Figure 13 shows the localization results in case 2. The drift error within a sub-edge is larger in case 2 because of the increasing sub-edge distance. Localization results in the asymmetrical light arrangement are shown in Figure 14 and Figure 15. The accumulative error can be eliminated at the first corner because of the timely modification after turning. However, the drift error of case 3 is larger than that of case 4 at the second corner owing to the larger distance between the second corner and the first ceiling-mounted lamp after turning.

The FirstFit approach matches the estimated heading direction with the orientation of the prescribed route. The drift error can be eliminated by providing the edge direction from the map. However, the incorrect step length estimation causes the accumulative error within the edge, as shown in Figure 16. If the estimated position reaches the end position of an edge, the estimated position will stop at the end of the edge until a turning situation is detected. The proposed fusion approach can correct the error of incorrect step length at the ending of each sub-edge to reduce the accumulative error of incorrect step length estimation. The results using the proposed system based on FirstFit is demonstrated in Figure 17, Figure 18, Figure 19 and Figure 20. With an appropriate light density in Figure 17, the estimated step length of each sub-edge is updated continually to reduce the overestimated (brown arrow) and underestimated (red arrow) step length situation. Even though there is a lower light density in Figure 18, the localization accuracy can be improved by modifying the position and step length. Figure 19 and Figure 20 demonstrate the localization results in asymmetrical light arrangement environments. The step length error of case 4 is larger than that of case 3 in edge 1. However, the localization accuracy is both good in case 3 and case 4 at the first corner because of the correct step length modification. At the second corner, the drift error of the gyroscope leads to a misidentified turning event. Despite the step length modification and pedestrian position revision, the error cannot be eliminated because of the different step length while turning. The error at the second corner should be tackled while the subject passes through the lamp after the second corner. The distance between the second corner and the next lamp may influence the error in the sub-edge.

**Figure 21:**
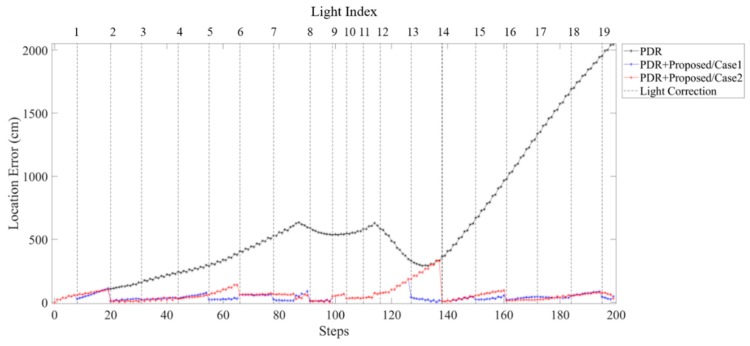
Comparison of error distance of each steps using PDR and proposed fusion approach in case 1 and case 2 light arrangement.

**Figure 22:**
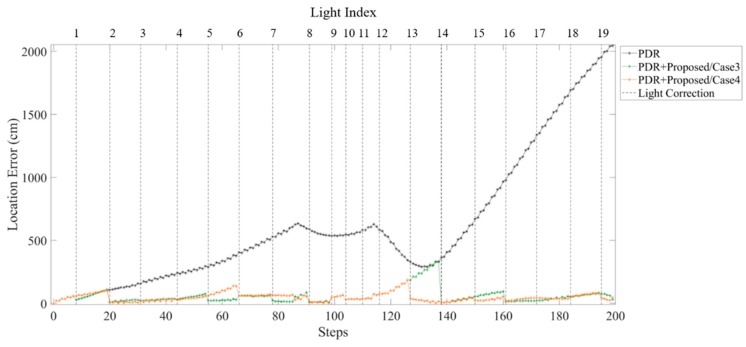
Comparison of error distance of each steps using PDR and proposed fusion approach in case 3 and case 4 light arrangement.

Comparison of error distance in PDR approach and proposed fusion approach in different light arrangements are shown in Figure 21 and Figure 22. The error distance of the PDR accumulated with steps can be observed. In Figure 21, both case 1 and case 2 can reduce the accumulative error at each sub-edge. However, case 2 which has a smaller light density reveals a larger location error from turning at L12 to L14. This localization error can be modified by the light event detection at L14. Figure 22 compares the location error of each step in case 3 and case 4. Before L8, the light density of case 3 is larger than that of case 4. The location error reveals smaller at case 3 but there is little difference between case 3 and case 4. Nevertheless, after turning at L12, the fewer light in case 3 causes accumulated error at L12 to L14 because of the larger sub-edge distance.

**Figure 23:**
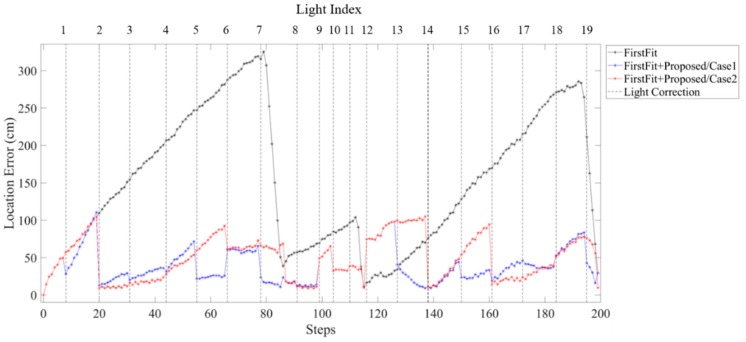
Comparison of the error distance of each steps using the First Fit approach and proposed fusion approach in case 1 and case 2 light arrangement.

**Figure 24:**
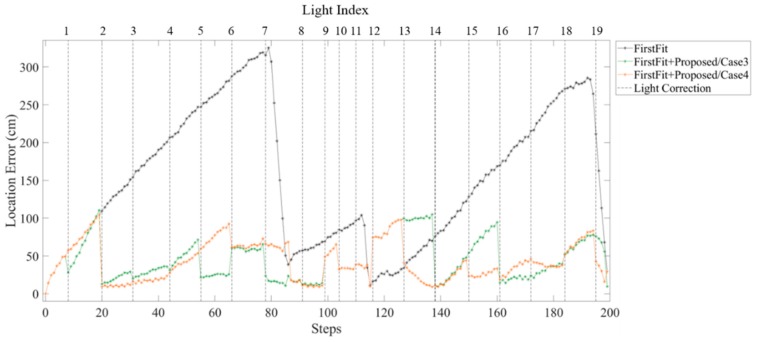
Comparison of the error distance of each steps using the First Fit approach and proposed fusion approach in case 3 and case 4 light arrangement.

Figure 23 and Figure 24 show the location error of each step using FirstFit and proposed fusion approach in different light environments. The error distance can be modified at each edge by FirstFit. However, within the edge, the error accumulated with steps. By fusing the light information, the location error of case 1 and case 2 as Figure 23 can be corrected within each sub-edge. The drift error after the second corner at L12 in case 2 is still large. In Figure 24, because of the higher light density before L8 in case 3, the location error of case 3 is a little smaller than that of case 4. However, the drift error that happened at the second corner may not be corrected in time due to the lower light density after L12 in case 3.

The average accumulative error of subjects by different approaches and light arrangement is illustrated in Table 1. The PDR approach reveals the worst localization accuracy with 430.2 cm average error distance. The accumulative error of the FirstFit approach is 160 cm owing to the improper step length estimation. The proposed fusion approach based on PDR can reduce the location error to 40.36 cm in case 1, 62.76 cm in case 2, 55.88 cm in case 3 and 49.23 cm in case 4. Moreover, the proposed system utilizing FirstFit can improve the error distance to 37.41 cm in case 1, 45.5 cm in case 2, 42.16 cm in case 3 and 40.75 cm in case 4. The results show that the multi-sensor fusion approach is feasible to improve the localization accuracy and the light density in the environment may influence the location accuracy.

## 5. Discussion

The main purpose of this study is developing a multi-sensor fusion approach by inertial sensors, light sensor and Bluetooth to improve the performance of a map-based indoor pedestrian localization system. The inertial sensor-based PDR system has advantages including no additional infrastructure needed and no radio frequency interference but has disadvantages including the error generated by drift error and inaccurate step length estimation that may be accumulated during walking. Previous studies utilize the Kalman filter and particle filter to improve the PDR system fusing with other technologies such as Wi-Fi, Bluetooth and light sensors. We present a simple approach by applying Bluetooth, light sensor and map information to deal with the issue of accumulative error in the PDR system. The proposed multi-sensor fusion approach is developed based on the information of the known route in an environment. Pedestrians may follow the planning route from an indoor navigation system to find their destination. On the basis of the planning route, the heading and light information along the planning route can be obtained. The light sensor and Bluetooth of a smartphone can detect the lamp and pre-installed beacons while a pedestrian passes under ceiling-mounted lamps and provide edge and sub-edge information. Then, the pedestrian position information can be inferred by comparing edge and sub-edge information on maps. With the position information, we can modify the individual step length, reset the sub-edge heading and revise the current position.

The results of the experiments demonstrate the feasibility of the proposed multi-sensor fusion approach. It reduces the accumulative error and improves the localization accuracy by fusing multi-sensor data from inertial sensors, the light sensor, Bluetooth and map. The drift error and incorrect step length estimation lead to an accumulative error of 430.20 cm in PDR. To reduce the drift error, the FirstFit approach diminishes the accumulative error to 160 cm by matching the heading direction of each step with the orientation of known route information to check for the turning events. The proposed multi-sensor fusion approach utilizes Bluetooth and the light model to identify edge and sub-edge information. The accumulative error of the proposed multi-sensor fusion approach in an appropriate light density environment can be reduced to 40.36 cm based on the PDR and 37.41 cm based on the FirstFit approach.

We designed four different light arrangements to evaluate the performance of the proposed fusion approach in different ceiling-mounted lamp conditions. According to the experimental results, there are two findings in this study. Firstly, light density in an environment may affect the localization accuracy. Compared to lower light density in case 2, the localization accuracy of higher light density in case 1 is better in both proposed fusion approaches based on PDR and FirstFit. Appropriate light density in the environment can reduce accumulative error effectively by edge and sub-edge information. As the light density decreases, the accumulative error increases. Secondly, a larger distance between a corner and the first ceiling-mounted lamp after a corner may cause a larger accumulative error. The distance from the first corner to the last ceiling-mounted lamp before corner in case 4 is larger than that in case 3. Although the light density of case 4 is lower than that of case 3 before the first corner, the accumulated error can be corrected by detecting the first ceiling-mounted lamp after the first corner. In contrast, the distance from the second corner to the first ceiling-mounted lamp after the corner in case 3 is larger than that in case 4. Despite the modification before turning, the drift error influences the localization after the second corner. Because of a larger distance between the second corner and the first ceiling-mounted lamp after the corner, the accumulative error is larger in case 3 than that in case 4. Therefore, in an asymmetrical light density environment, larger distance between a corner and the first ceiling-mounted lamp after turning causes larger accumulative error.

The multi-sensor fusion approach can reduce the accumulated error effectively by providing edge and sub-edge information. However, there are still some limitations in this study. Firstly, the smartphone is required to face up for the detection of visible light. Nevertheless, while using a navigation system for wayfinding in an environment, the pedestrian may hold the smartphone facing up to check the instructions. In addition, inertial sensors are still working despite a lack of light information. If the smartphone cannot obtain light information, the localization can be estimated by the traditional PDR or Footpath approach. The misidentified light event caused by burnt-out lamps or smartphone position can be detected by matching the expected walking distance between two lamps. The walking distance of the pedestrian can be estimated by step detection and step length estimation. If the walking distance is much longer than the distance between two lamps, we can infer that there may be a missed lamp among the route and utilize information of the next lamp for decision making. Secondly, the determination of the initial position is not considered in this study. We focus on solving the issue of accumulative error rather than initial position. There are many studies utilizing different technologies to provide initial location. Buti Al Delail et al. [34] utilize an image marker by camera to provide initial location. Chen et al. [44] combine Wi-Fi and Bluetooth to obtain the initial location. Fuqiang et al. [53] employ quick response (QR) code as landmarks for location. Based on the studies mentioned above, the issue of initial location can be tackled by using QR code, NFC or image markers. Moreover, with the planning route of navigation system, the initial heading direction can be assumed to the initial instruction of navigation system.

## 6. Conclusions

To improve the accuracy of indoor pedestrian localization, we propose a multi-sensor fusion approach using inertial sensors, light sensor, Bluetooth and map information in this study. Compared to the traditional PDR system and FirstFit approach, the proposed multi-sensor fusion approach can improve 90% and 76% of localization accuracy in an environment with appropriate light density, respectively. The accumulative error of proposed multi-sensor fusion approach is below 65 cm in different cases of light arrangement. Although the localization accuracy may be influenced by light density in the environment, the accumulative error is much lower than the PDR and FirstFit in each case. According to the results, the proposed multi-sensor fusion approach is feasible to reduce the accumulative error and improve the localization accuracy by combination of inertial sensors, light sensor, Bluetooth and map information. The step length modification, sub-edge heading reset and pedestrian position revision can reduce the accumulative error effectively. In future work, we plan to apply the multi-sensor fusion approach in complex environment that includes routes with multiple branches and improve the robustness of proposed multi-sensor fusion approach.

## Figures and Tables

**Table 1:** The average accumulative error of subjects with different approaches and light arrangement.

	PDR	PDR-Proposed	FirstFit	FirstFit-Proposed
Case 1	430.20 ± 138.90	40.36 ± 7.64	160.00 ± 38.30	37.41 ± 6.62
Case 2		62.76 ± 12.52		45.50 ± 9.17
Case 3		55.88 ± 13.13		42.16 ± 8.19
Case 4		49.23 ± 7.91		40.75 ± 7.97

Unit: cm.
